# Development of a predictive model for postoperative body mass index and diabetes outcomes after metabolic bariatric surgery: retrospective cohort study

**DOI:** 10.1093/bjsopen/zrag054

**Published:** 2026-07-03

**Authors:** Vincent Ochs, Lars Kollmann, Ilan Rosenblum, Adisa Poljo, Andreas Heule, Bassey Enodien, Maryna Chumakova-Orin, Eric J DeMaria, Emanuel Burri, Reinhard Stoll, Otto Kollmar, Robert Rosenberg, Pascal Probst, Markus K Muller, Stephanie Taha-Mehlitz, Beat P Müller, Daniel M Frey, Piotr Kalinowski, Marta Przybysz, Mateusz Bartkowiak, Muhammed Said Dalkilic, Abdullah Sisik, Rodrigo Otavio Carvalho De Oliveria, Fatima Martins, Erik Stenberg, Ellen Andersson, Torsten Olbers, Sven Flemming, Florian Seyfried, Florian Ponholzer, Annemarie Weissenbacher, Dietmar Öfner, Johanna Betzler, Mirko Otto, Ralph Peterli, Philippe C Cattin, Anas Taha

**Affiliations:** Department of Biomedical Engineering, Faculty of Medicine, University of Basel, Basel, Switzerland; Department of General, Visceral, Transplantation, Vascular, and Pediatric Surgery, University Hospital Wuerzburg, Wuerzburg, Germany; Department of Visceral Surgery, Cantonal Hospital Baselland, Liestal, Switzerland; Clarunis, Department of Visceral Surgery, University Center for Gastrointestinal and Liver Diseases, St.Clara Hospital and University Hospital Basel, Basel, Switzerland; Johannes Kepler University Linz, Medical Faculty, Linz, Austria; Department of Biomedical Engineering, Faculty of Medicine, University of Basel, Basel, Switzerland; Institute for Medical Informatics, University of Applied Sciences of Northwestern Switzerland, Muttenz, Switzerland; Department of Surgery, Cantonal Hospital Glarus, Glarus, Switzerland; Department of Surgery, East Carolina University, Brody School of Medicine, Greenville, North Carolina, USA; Department of Surgery, East Carolina University, Brody School of Medicine, Greenville, North Carolina, USA; University Institute of Internal Medicine, Cantonal Hospital Baselland, Liestal, Switzerland; Clarunis, Department of Visceral Surgery, University Center for Gastrointestinal and Liver Diseases, St.Clara Hospital and University Hospital Basel, Basel, Switzerland; Clarunis, Department of Visceral Surgery, University Center for Gastrointestinal and Liver Diseases, St.Clara Hospital and University Hospital Basel, Basel, Switzerland; Clarunis, Department of Visceral Surgery, University Center for Gastrointestinal and Liver Diseases, St.Clara Hospital and University Hospital Basel, Basel, Switzerland; Department of Surgery, Cantonal Hospital Thurgau, Frauenfeld, Switzerland; Department of Surgery, Cantonal Hospital Thurgau, Frauenfeld, Switzerland; Johannes Kepler University Linz, Medical Faculty, Linz, Austria; Johannes Kepler University Linz, Medical Faculty, Linz, Austria; Department of Surgery, Kantonspital Baden, Baden, Switzerland; Department of General, Transplant and Liver Surgery, Medical University of Warsaw, Warsaw, Poland; Department of General, Transplant and Liver Surgery, Medical University of Warsaw, Warsaw, Poland; Department of General, Transplant and Liver Surgery, Medical University of Warsaw, Warsaw, Poland; Department of General Surgery, Marmara University Faculty of Medicine, Istanbul, Turkey; Department of General Surgery, Marmara University Faculty of Medicine, Istanbul, Turkey; iNOVA4Health, NOVA Medical School, Faculty of Medical Sciences, NOVA University of Lisbon, Lisbon, Portugal; iNOVA4Health, NOVA Medical School, Faculty of Medical Sciences, NOVA University of Lisbon, Lisbon, Portugal; Department of Surgery, Faculty of Medicine and Health, Örebro University, Örebro, Sweden; Department of Surgery and Department of Clinical and Experimental Medicine, Linköping University, Norrköping, Sweden; Department of Surgery and Department of Clinical and Experimental Medicine, Linköping University, Norrköping, Sweden; Department of General, Visceral, Transplantation, Vascular, and Pediatric Surgery, University Hospital Wuerzburg, Wuerzburg, Germany; Department of General, Visceral, Transplantation, Vascular, and Pediatric Surgery, University Hospital Wuerzburg, Wuerzburg, Germany; Department of Visceral, Transplant and Thoracic Surgery, Center of Operative Medicine, Medical University of Innsbruck, Innsbruck, Austria; Department of Visceral, Transplant and Thoracic Surgery, Center of Operative Medicine, Medical University of Innsbruck, Innsbruck, Austria; Department of Visceral, Transplant and Thoracic Surgery, Center of Operative Medicine, Medical University of Innsbruck, Innsbruck, Austria; Department of Surgery, University Medical Center Mannheim, Medical Faculty Mannheim, Heidelberg University, Mannheim, Germany; Department of Surgery, University Medical Center Mannheim, Medical Faculty Mannheim, Heidelberg University, Mannheim, Germany; Department Clinical Research, University of Basel, Basel, Switzerland; Department of Biomedical Engineering, Faculty of Medicine, University of Basel, Basel, Switzerland; Department of Biomedical Engineering, Faculty of Medicine, University of Basel, Basel, Switzerland; Department of Visceral Surgery, Cantonal Hospital Baselland, Liestal, Switzerland; Department of Surgery, East Carolina University, Brody School of Medicine, Greenville, North Carolina, USA

**Keywords:** machine learning, diabetes remission, postoperative weight loss, risk prediction

## Abstract

**Background:**

Predicting postoperative body mass index (BMI) trajectories and long-term type 2 diabetes (T2D) remission after bariatric surgery remains challenging. Existing models often rely on baseline variables only and fail to incorporate dynamic postoperative changes. This study aimed to develop and validate a multicentre machine-learning framework that predicts individualized BMI trajectories and T2D remission using routinely available preoperative data and time-dependent weight evolution.

**Methods:**

This multicentre retrospective cohort study included adult patients who underwent Roux-en-Y gastric bypass or sleeve gastrectomy across 11 European centres (2012–2023). Variables with > 30% missing data were excluded; remaining missing values were imputed iteratively. A two-stage approach was used: a regression model predicting postoperative BMI at 3–60 months using an autoregressive design; and a classification model predicting T2D remission using baseline features and predicted BMI trajectories. Internal performance was evaluated with ten-fold and leave-one-clinic-out cross-validation; external validation used an independent cohort from Linköping, Sweden.

**Results:**

Of the 11 457 patients initially identified, 9652 patients with complete baseline and follow-up information were used for the analysis. The best BMI model (HistGradientBoosting) achieved a root mean square error (RMSE) of 1.11 kg/m^2^ (95% confidence interval 1.07 to 1.14) and a mean absolute error (MAE) of 0.62 kg/m^2^ across clinics; external testing showed an RMSE of 1.12 kg/m^2^ (95% confidence interval 1.11 to 1.12) and an MAE of 0.63 kg/m^2^. The T2D remission classifier (XGBoost) obtained a Macro F1 score of 0.88 (precision 0.87, recall 0.88), with an external F1 score of 0.89. Incorporating predicted BMI trajectories improved discrimination compared with baseline-only models (C-index 0.95 *versus* 0.93).

**Conclusion:**

A two-stage machine-learning framework has high predictive performance for postoperative BMI and T2D remission up to 5 years after bariatric surgery. Dynamic incorporation of predicted weight trajectories enhances metabolic risk prediction and supports individualized counselling and postoperative management.

## Teaser

A two-stage, autoregressive machine-learning framework to predict postoperative body mass index trajectories and type 2 diabetes remission following metabolic bariatric surgery was developed and externally validated. Using multicentre registry data from 9652 patients across 11 centres and an independent external cohort, the model demonstrated accurate and robust prediction of long-term weight evolution and diabetes outcomes. This approach enables time-aware, clinically realistic risk stratification to support individualized postoperative counselling and follow-up.

## Introduction

The global prevalence of obesity has increased considerably over past decades, becoming a significant public health concern^1,2^. Obesity is a multifactorial chronic disease associated with many obesity-related conditions that can decrease life expectancy^3^. Metabolic and bariatric surgery (MBS) has emerged as the most effective intervention for achieving substantial and sustainable weight loss while improving obesity-related co-morbidities and prolonging life expectancy^4^. Many studies^4,5^ have described the positive effects of MBS.

Despite preoperative assessment and previous attempts to develop prediction models^6^, individual outcomes after MBS, particularly long-term body mass index (BMI) and type 2 diabetes (T2D0 trajectories, remain difficult to predict.

Identifying preoperative factors that influence and could predict the BMI trajectory and T2D remission would guide preoperative decision-making and offer realistic expectancy for individualized treatment strategies.

Factors such as age, sex, the severity of diabetes, medication history, previous weight loss attempts, and psychosocial characteristics have been implicated in predicting weight loss and metabolic improvement^7–9^. However, traditional statistical models have struggled to integrate multidimensional patient profiles into individualized predictions of postoperative outcomes^10^, and to create prediction models for both BMI and T2D for different surgical approaches.

Machine learning methods offer the advantage of identifying intricate patterns within data that may be difficult to capture using conventional statistical techniques. In particular, ensemble methods such as XGBoost, random forest, and HistGradientBoosting, which combine the predictions of many smaller models to achieve more robust and accurate results, have shown promising performance in complex healthcare prediction tasks^11,12^. These models excel in capturing non-linear dependencies and feature interactions, making them well-suited for predicting BMI progression and metabolic outcomes after MBS^13,14^.

Recently, some studies developed machine learning algorithms to predict MBS outcomes in large patient cohorts^15,16^. These models achieved high predictive accuracy for weight and BMI trajectories up to 5 years after MBS, with a favourable mean absolute deviation compared with retrospectively assessed values.

Data quality remains challenging for the learning process and the prospective evaluation of the prediction.

To date, no model has been developed using machine learning methods to predict the longitudinal course of T2D after sleeve gastrectomy (SG) or Roux-en-Y gastric bypass (RYGB). Thus, in the present study, a two-stage machine learning framework was leveraged to build a model for BMI and T2D prediction after metabolic and bariatric surgery.

## Methods

### Data collection

Data were retrospectively collected from 11 European bariatric centres, including national registries and institutional databases (*supplementary methods*). This study was completed based on the TRIPOD statement checklist for developing clinical prediction models (*Table S1*)^17^.

This study involved human participants and was approved by the Northwest- and Central Switzerland Ethics Committee EKNZ, Switzerland (BASEC-Nr 2022-00659).

### Patient data

Adult (age ≥ 18 years) patients who underwent primary RYGB or SG and had complete preoperative data and a complete follow-up for at least 1 year were eligible for inclusion in the study. A flow chart of the data selection process is provided in *Fig. S1*.

Clinical patient variables extracted for this study included demographic characteristics, co-morbidities, and procedural details. Demographic variables included sex (female, male), BMI, and age. Additional clinical factors considered included the American Society of Anesthesiologists score, the Charlson co-morbidity index, previous abdominal surgery, hypertension, hyperlipidaemia, depression, and antidepressant medications. Diabetes-related data included T2D (preoperative and postoperative), as well as detailed records on antidiabetic medication use (dietary, oral antidiabetic drugs, insulin, no therapy, glucagon-like peptide-1 analogues). Data on obstructive sleep apnoea syndrome, both before and after bariatric surgery, was also included.

### Surgical data

Surgical factors included the type and duration of surgery and conversions from SG to RYGB. Postoperative complications were classified according to the Clavien–Dindo system (grades I–V), with complications identified during follow-up (internal hernia, hiatal hernia, GERD, ulcers) also assessed^18^. BMI and T2D progression were tracked at multiple timepoints up to 5 years (*Table S2*). The characteristics of patients in the external validation cohort (Linköping) are provided in *Table S3*.

Variables with excessive missing data were excluded to limit bias from extensive imputation. A threshold of 30% missing data (*Tables S2* and *S3*) was selected based on a sensitivity analysis evaluating thresholds between 10 and 50%, which demonstrated stable model performance across this range, with optimal performance observed at the 30% cut-off. Remaining missing data were imputed iteratively^19^. Continuous variables were normalized, categorical variables were one-hot encoded^20^, and polynomial feature expansion was applied to capture non-linear interactions^21^.

### Model training

The proposed framework is an autoregressive two-stage prediction pipeline rather than a formal joint longitudinal model. The first stage trained a regression model to predict postoperative BMI sequentially using an autoregressive approach. BMI at 3 months was predicted from preoperative features, whereas later time points incorporated both baseline variables and previously predicted BMI values as dynamic covariates. The model was trained within a single unified framework covering all postoperative intervals (3, 6, 9, 12, 24, 36, 48, and 60 months), thereby leveraging temporal dependencies without training separate models per interval. This design mimics real-world clinical deployment, where each new follow-up refines previous estimates and enables continuous, time-aware prediction of postoperative weight trajectories.

Algorithms evaluated included XGBoost, random forest, HistGradientBoosting, and a Multilayer Perceptron, with the best-performing model generating individualized BMI predictions.

In the second stage, a classification model predicted T2D remission based on preoperative characteristics and predicted BMI trajectories. Remission was defined according to the international consensus by Riddle *et al*.^22^ (that is, HbA1c < 6.5% maintained for at least 3 months without antidiabetic medication). In practice, remission status was provided by each participating hospital as a documented binary field (remission achieved or not achieved) within their bariatric registry. Although the hospitals used the consensus definition, individual HbA1c values were not consistently available across all centres. Consequently, remission was operationalized based on the recorded diabetes status and the absence of antidiabetic medication at each follow-up visit. Ensemble methods were applied, and performance was assessed by the Macro F1 score^23–25^.

The diabetes remission model was formulated as a binary classification problem (remission *versus* no remission at each timepoint). It used baseline features and corresponding predicted BMI values up to the same follow-up timepoint as input predictors. Importantly, only BMI predictions from the same or earlier timepoints were used when predicting remission, ensuring that the model relied solely on information that would be available at the time of prediction. This approach prevents any temporal data leakage and preserves the model’s prospective validity. These models were not survival (time-to-event) models but rather independent classification estimators for remission status at each timepoint.

### Temporal alignment and the use of predicted *versus* observed BMI

For each postoperative timepoint, predictions were generated using only information that would be available up to that timepoint. When predicting BMI at a given timepoint, the regression model used baseline features and previously predicted BMI values from earlier intervals, but never future BMI values. The diabetes remission classifier used baseline variables and the corresponding predicted BMI values up to the same timepoint. Observed BMI values were used exclusively as outcome labels for training and evaluation, but not as inputs at inference time, preventing temporal data leakage.

### Refinement

To further refine the approach, robust preprocessing steps were implemented, including the SMOTE-Tomek technique to oversample minority cases and remove noisy data points^26,27^ (*supplementary methods*).

Model evaluation involved rigorous ten-fold cross validation to ensure robust performance across diverse patient groups. The best-performing models were further refined using a grid search for hyperparameter tuning (*Table S5*). Performance was assessed using root mean square error (RMSE) and mean absolute error (MAE) for BMI predictions, whereas classification performance was measured using the F1 score, to ensure reliable and clinically meaningful predictions^14,15^. The RMSE quantifies the mean magnitude of prediction error in the same unit as the outcome (kg/m^2^); for example, an RMSE of 3 kg/m^2^ indicates that the predicted BMI typically differs from the true postoperative BMI by approximately 3 kg/m^2^. MAE provides a more robust measure of typical deviation, being less influenced by outliers. For the binary classification task predicting T2D remission, accuracy, precision, recall, and Macro F1 score are reported. Precision indicates the proportion of patients predicted as remitted who truly achieved remission, recall reflects the proportion of all remitted patients correctly identified, and the F1 score summarizes the balance between both.

### Validation

Model validation combined complementary internal and external strategies to ensure robustness and generalizability. Ten-fold cross-validation was used for internal performance estimation, whereas a leave-one-clinic-out procedure served as an internal–external validation strategy, assessing generalization across participating centres within the multicentre registry. In addition, the Linköping (Sweden) cohort (2356 patients), which was entirely excluded from model training and tuning, was used as a fully independent external validation data set. Detailed validation procedures, including per-clinic rotation and external benchmarking, are described in the *supplementary methods*. For regression performance, RMSE and MAE are reported as mean values with their corresponding 95% confidence interval (c.i.), computed across validation folds in the leave-one-clinic-out cross-validation. Confidence intervals reflect variability in model performance due to differences in training data composition across centres.

To evaluate the clinical utility of the diabetes remission model, a decision curve analysis was performed (*Fig. S2*). To benchmark against classical statistical approaches, Cox proportional hazards models were fitted using baseline variables alone and with observed postoperative BMI as a time-varying covariate (*Fig. S3*). To further interpret model behaviour, permutation-based feature importance analyses were conducted for both the regression and classification stages (*Fig. S4* and *S5*).

### Web-based application

Following model development, a free-to-use web-based application was deployed to facilitate real-world clinical use in individual cases. The application can be accessed at https://diabetesmellitus.vercel.app.

## Results

Of the 11 457 patients initially identified, 9652 patients with complete baseline and follow-up information were used for the analysis (*Fig. S1*). For the 12- and 18-month and 2-, 3-, 4-, and 5-year timepoints, data were available for 9652, 6555, 3129, 1915, 971, and 441 patients, respectively.

Model performance metrics are summarized in *Table 1*. The best-performing regression model (HistGradientBoosting) achieved a mean RMSE of 1.11 kg/m^2^ (95% c.i. 1.07 to 1.14) and an MAE of 0.62 kg/m^2^ (95% c.i. 0.61 to 0.63) across leave-one-clinic-out validation folds. On the external Linköping test set, performance remained nearly identical (RMSE, 1.12 kg/m^2^; MAE, 0.63 kg/m^2^). This corresponds to a mean deviation of one BMI unit (that is, 1 kg/m^2^) between predicted and observed postoperative values.

**Table 1:** Regression performance evaluation of all models used (for the mean over ten-fold cross-validation and across all clinics and timepoints) for the prediction of postoperative BMI

Model	Metric	Validation	
		Cross-validation	External test set
XGBoost	RMSE	1.12 (1.08, 1.15)	1.13 (1.13, 1.13)
	MAE	0.63 (0.62, 0.64)	0.64 (0.64, 0.64)
	MAPE	24.17 (24.05, 24.29)	23.98 (23.97, 23.98)
Random Forest	RMSE	1.15 (1.11, 1.18)	1.13 (1.13, 1.14)
	MAE	0.64 (0.63, 0.65)	0.64 (0.64, 0.64)
	MAPE	24.16 (24.04, 24.28)	23.97 (23.97, 23.98)
HistGradientBoosting	RMSE	1.11 (1.07, 1.14)	1.12 (1.11, 1.12)
	MAE	0.62 (0.61, 0.63)	0.63 (0.63, 0.63)
	MAPE	24.14 (24.02, 24.26)	23.96 (23.95, 23.96)
MLP	RMSE	1.83 (1.70, 1.97)	1.83 (1.72, 1.95)
	MAE	1.33 (1.21, 1.45)	1.33 (1.21, 1.44)
	MAPE	23.93 (23.42, 24.43)	23.76 (23.32, 24.21)

Values in parentheses are 95% confidence intervals. Regression performance for predicting postoperative BMI across all timepoints using different machine-learning algorithms is shown. Model performance was assessed by the RMSE, MAE, and MAPE. Internal validation was conducted with a leave-one-clinic-out approach across all participating centres except Linköping, which was reserved as a completely independent external test set. Lower RMSE and MAE values indicate higher predictive accuracy. Among all tested models, HistGradientBoosting achieved the lowest RMSE and MAE during cross-validation and maintained similar performance on the external Linköping data set (RMSE, 1.12; MAE, 0.63), confirming excellent generalization. XGBoost and random forest performed comparably, whereas the MLP showed higher error variability. The narrow confidence intervals across models demonstrate low variance and strong robustness of the regression framework, consistent with the results reported in *Tables 2* and *3*. BMI, body mass index; RMSE, root mean square error; MAE, mean absolute error; MAPE, mean absolute percentage error; MLP, multilayer perceptron.

Performance across clinics was consistent, with a mean RMSE of 1.11 kg/m^2^ (range 0.82–1.51 kg/m^2^), demonstrating low inter-clinic variability (*Table 2*). Calibration analysis revealed no systematic bias across prediction intervals (*Fig. S6*).

**Table 2:** Performance evaluation of the best model used (for the mean ten-fold cross-validation across all clinics and timepoints) to predict postoperative BMI

Clinic	RMSE (kg/m^2^)							
	Month 3	Month 6	Month 12	Month 18	Year 2	Year 3	Year 4	Year 5
Innsbruck	2.13	1.27	1.46	1.21	0.94	0.57	1.04	1.17
Warsaw	2.7	0.89	0.76	0.87	0.97	1.01	1.17	1.21
Würzburg	1.76	1.4	1.66	1.67	0.63	1.21	0.9	0.98
NOVA Lisbon	1.87	1.49	1.13	1.96	0.94	0.75	0.56	0.89
Mannheim	2.55	0.74	0.72	0.53	0.41	0.42	0.7	0.89
Clarunis	2.21	0.74	0.74	0.55	0.4	0.41	0.68	0.98
Thurgau	2.43	0.79	0.78	0.57	0.4	0.76	0.63	0.71
GZO Wetzikon	3.94	2.55	0.72	0.59	0.41	0.6	0.65	0.85
KSBL Liestal	2.14	0.57	0.57	0.48	0.41	0.72	0.78	0.96
Marmara Istanbul	1.89	2.66	0.61	0.56	0.76	1.38	2.09	2.14
Mean	2.36	1.31	0.91	0.90	0.62	0.78	0.92	1.01

RMSE values quantifying prediction accuracy for postoperative BMI across different clinical sites and timepoints are shown. Internal validation was performed with a leave-one-clinic-out strategy across all participating centres except Linköping, which was held out as a completely independent external test cohort (see *Table 1*). The RMSE represents the mean deviation between predicted and observed BMI (in kg/m^2^), with lower values indicating higher predictive accuracy. Predictions were evaluated at 3, 6, 12, and 18 months and annually up to 5 years after surgery. Overall, the HistGradientBoosting model achieved consistently low BMI errors across centres (typically around 1.11 kg/m^2^). The small interclinic variability and narrow range of RMSE values demonstrate good generalization of the regression framework across heterogeneous clinical environments. Weight loss trajectories derived from predicted BMI values showed the expected rapid decline within the first 12 months followed by a long-term stabilization phase. BMI, body mass index; RMSE, root mean square error.

**Fig. 1:**
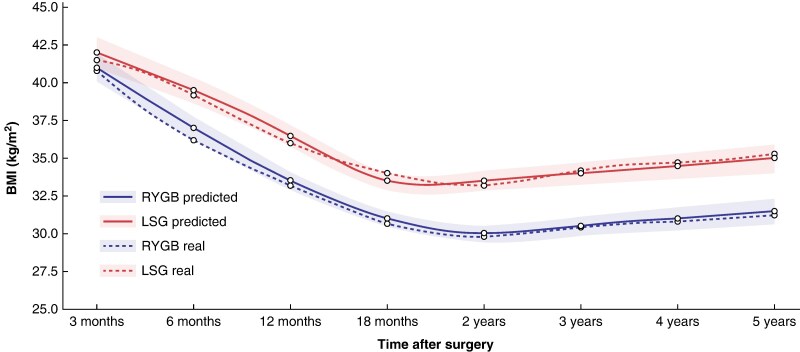
Predicted *versus* real BMI trajectories over 5 years for the two different surgical interventions Solid lines represent the predicted BMI values; shaded areas indicate 95% confidence intervals. Dashed lines represent the real (observed) BMI values from the data set. RYGB showed the most favourable reduction in BMI. The confidence intervals highlight the prediction uncertainty, and the close alignment of predicted and real curves indicates good model performance in approximating postoperative BMI trends. BMI, body mass index; RYGB, Roux-en-Y gastric bypass; LSG, laparoscopic sleeve gastrectomy.

A comparison of predicted *versus* observed BMI curves for the two surgical interventions is shown in *Fig. 1*. Stratified results by timepoint are reported in *Table 3*.

**Table 3:** Evaluation of the classification performance of all models used (for the mean over ten-fold cross-validation across all clinics and timepoints) for predicting diabetes remission

Model	Metric	Validation	
		Cross-validation	External test set
XGBoost	Accuracy	0.97 (0.97, 0.97)	0.98 (0.98, 0.98)
	Precision	0.87 (0.86, 0.88)	0.88 (0.88, 0.88)
	Recall	0.88 (0.87, 0.89)	0.90 (0.89, 0.90)
	F1 score	0.88 (0.87, 0.88)	0.89 (0.89, 0.89)
	AUC_ROC_	0.99 (0.99, 0.99)	1.00 (0.99, 1.00)
	Log Loss	0.07 (0.06, 0.07)	0.06 (0.06, 0.06)
Random forest	Accuracy	0.96 (0.95, 0.96)	0.96 (0.96, 0.96)
	Precision	0.78 (0.77, 0.79)	0.79 (0.79, 0.79)
	Recall	0.82 (0.81, 0.84)	0.84 (0.84, 0.84)
	F1 score	0.80 (0.79, 0.81)	0.81 (0.81, 0.82)
	AUC_ROC_	0.98 (0.98, 0.99)	0.99 (0.99, 0.99)
	Log loss	0.15 (0.14, 0.17)	0.13 (0.13, 0.14)
HistGradientBoosting	Accuracy	0.97 (0.97, 0.97)	0.97 (0.97, 0.97)
	Precision	0.85 (0.84, 0.86)	0.86 (0.86, 0.87)
	Recall	0.88 (0.87, 0.89)	0.89 (0.89, 0.89)
	F1 score	0.87 (0.86, 0.87)	0.88 (0.88, 0.88)
	AUC_ROC_	0.99 (0.99, 0.99)	0.99 (0.99, 0.99)
	Log loss	0.07 (0.07, 0.07)	0.06 (0.06, 0.06)
MLP	Accuracy	0.87 (0.77, 0.96)	0.87 (0.77, 0.96)
	Precision	0.58 (0.42, 0.74)	0.58 (0.42, 0.75)
	Recall	0.90 (0.84, 0.96)	0.90 (0.84, 0.97)
	F1 score	0.67 (0.54, 0.79)	0.67 (0.54, 0.80)
	AUC_ROC_	0.98 (0.97, 0.98)	0.98 (0.97, 0.98)
	Log loss	1.06 (0.18, 1.93)	1.06 (0.15, 1.97)

Values in parentheses are 95% confidence intervals. This table presents the classification performance evaluation for predicting diabetes remission using various machine learning models. Performance was assessed via ten-fold cross-validation across all clinics and timepoints, using five standard classification metrics, and on the external test set. Accuracy is defined as the proportion of correct predictions of all predictions. Precision is defined as the proportion of true positives among predicted positives. Recall (sensitivity) is defined as the proportion of true positives correctly identified. The AUC_ROC_ quantifies the model’s overall discrimination ability across all possible classification thresholds, with a value of 1 indicating perfect discrimination between remission and non-remission, whereas a value of 0.5 indicates random performance. Log loss (cross-entropy loss) is a measure of how well the predicted probabilities match the true labels. Log loss penalizes confident but incorrect predictions more heavily than less certain ones, with lower values indicating better calibrated and more reliable probability estimates. The F1 score is the harmonic mean of precision and recall, balancing both. AUC_ROC_, area under the receiver operating characteristic curve.

**Fig. 2:**
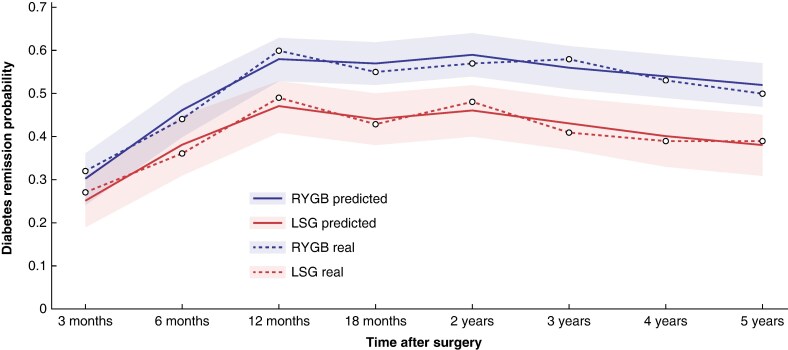
Probability of diabetes remission stratified by surgical intervention The probability of predicted *versus* real diabetes remission over 5 years is shown for two types of bariatric surgery (RYGB, LSG). Solid lines represent the model-predicted remission probabilities; shaded areas indicate 95% confidence intervals. Dashed lines indicate the observed (real) remission rates in the patient data set. The figure highlights that RYGB consistently shows the highest remission probability over time. After peaking around 12–24 months, the remission probabilities decline slightly across both surgery types, reflecting realistic clinical patterns of partial diabetes recurrence over long-term follow-up. RYGB, Roux-en-Y gastric bypass; LSG, laparoscopic sleeve gastrectomy.

The influence of surgical type on diabetes remission is shown in *Fig. 2*, and the effect of age on remission probability is shown in *Fig. S7*.

In the second modelling stage, a classification model predicted T2D remission at each follow-up timepoint using both preoperative variables and dynamically predicted BMI trajectories. The best-performing classifier (XGBoost) achieved a Macro F1 score of 0.88 (95% c.i. 0.87 to 0.88) across validation folds, with a precision of 0.87 and a recall of 0.88.

Performance on the independent Linköping test set remained stable (F1 = 0.89).

Clinic-specific F1 scores are reported in *Table S6*. Model performance stratified by surgical intervention is presented in *Tables S7* and *S8*.

## Discussion

This study demonstrates the development and validation of a machine learning framework for BMI and T2D remission prediction after metabolic and bariatric surgery. The model used preoperative variables, postoperative BMI timepoints, and metabolic outcomes. By combining ensemble regression and classification algorithms with predicted BMI trajectories, it achieved high accuracy and generalizability across clinical sites.

The framework uses a two-step design: first predicting BMI at multiple postoperative timepoints, then forecasting T2D remission from dynamic BMI changes and baseline factors. This staged approach better reflects the temporal complexity of metabolic change than single-step models from baseline data^6^.

One of the strengths of the two-step framework is its clinical applicability. The selected features are readily available in most clinical settings and do not require advanced diagnostic procedures. Other measures, such as diabetes duration, are also known to influence diabetes remission^28^. The machine learning algorithm integrates these variables with predicted BMI changes to provide the expected weight loss and T2D remission. This could enhance both preoperative counselling and postoperative monitoring.

The low variance of performance estimates across folds and clinics (as reflected by narrow confidence intervals in *Tables 1* and *3*) further supports the robustness of the models, even in the presence of moderate missing data and heterogeneous follow-up patterns. This stability arises from the ensemble-based architecture, which aggregates multiple weak learners, and thus mitigates sensitivity to data fluctuations and local biases, a limitation often seen in conventional regression or logistic models^29^.

Predicted BMI values contributed significantly to the classification of T2D remission outcomes, suggesting that weight loss mediates T2D remission. Interestingly, the model predicted precise values without further information about β-cell activity, as previous static prediction models needed. Incorporating these time-dependent features allowed the classifier to outperform static models (*Fig. S3*), highlighting the importance of integrating longitudinal follow-up dynamics in outcome prediction.

Importantly, the model does not rely on future or unseen follow-up data to make its predictions. Instead, it uses predicted BMI values generated by the first regression stage to represent expected weight trajectories over time. This design mirrors clinical reality, where each new follow-up provides additional information that refines the patient’s prediction. By simulating these temporal dynamics from preoperative and early postoperative data, the model captures realistic patterns of metabolic evolution without accessing future observations, thereby maintaining true prospective predictive capability. The model simulates a clinically realistic scenario by using previously predicted BMI values as input for subsequent timepoint predictions.

Deploying the model into a web-based interface adds to its utility. The interface enables simulation of patient-specific outcomes over time, aiding in surgical decision-making and the early identification of patients at risk of suboptimal results. This tool may allow re-evaluation of patient progress throughout follow-up.

The prediction capability of the model shows precise predictions regarding postoperative follow-up up to 5 years. The model demonstrates strong alignment between predicted and observed BMI, particularly during the first 12 months when weight loss is most pronounced (*Fig. 1*). The more substantial BMI reduction following RYGB than SG aligns with known physiological effects, such as hormone shifts promoting sustained weight loss.

Interestingly, beyond 18 months, the trajectories flatten across all procedures, representing the plateau phase after MBS. The divergence between predicted and observed BMI values around 18 months and 4 years likely reflects both physiological variability and reduced patient follow-up over an extended period. Behavioural factors such as dietary adherence and physical activity, not captured in the model, may further explain this variation. Adherence and compliance may have an impact, in addition to an individual’s biology. The model’s ability to forecast weight trends with reasonable accuracy highlights its robustness. Compared with previously published models, this model is able to predict BMI and T2D remission probability. Saux *et al*.^15^ were the first to develop a similar model to predict postoperative weight, intended as a patient-oriented, web-based tool with easily understood input parameters. However, the model developed in that study did not present diabetes remission probability. The present study did not include adjustable gastric banding at the point of conceptualization. In fact, recent studies report an inferior outcome for this procedure^28^, and its importance is further declining.

The close overlap between predicted and actual remission trajectories in the present study supports integrating dynamic, time-dependent BMI data as a meaningful predictor of T2D remission. In the present study, the model’s performance suggests that baseline factors do not solely determine remission probability but are intricately tied to postoperative weight dynamics, confirming weight loss as a major modifiable factor influencing metabolic recovery. Incorporating predicted BMI trajectories thus transforms remission prediction from a static to a dynamic longitudinal risk assessment tool. Although the model developed in this study enhances prediction accuracy, it cannot replace the need for clinical follow-up. The results underline the potential for misinterpretation if models generalize across procedures without adjustment, strengthening the argument for stratified, procedure-specific prediction pipelines in future work.

The model developed in this study does not incorporate socioeconomic, nutritional, or behavioural factors. In fact, the validation process from the SOPHIA trial^15^ already proved that only very few key parameters are needed to predict weight loss at a precise level, because machine learning models can calculate a prediction on recurrent patterns. This is possible because tree-based models, such as classification and regression trees, can capture non-linear relationships and interactions between highly informative features. The model reduces noise from less relevant variables and leverages recurrent decision patterns captured within the data set, enabling precise trajectory forecasting without requiring an extensive array of input variables.

Beyond metabolic and bariatric surgery, this study illustrates a general paradigm for surgical and clinical outcome prediction, in which dynamic postoperative trajectories are modelled rather than static endpoints. The proposed two-stage framework shows how baseline characteristics and early postoperative signals can support longitudinal risk estimation, underscoring the broader relevance of trajectory-based machine learning in clinical decision-making.

Future research should evaluate whether integrating dynamic prediction models into clinical decision support systems leads to improved outcomes compared with standard follow-up strategies.

This study has some limitations. The underlying data sets originate from different levels of study quality, partly from retrospective collection. This may impair data quality, and therefore may have impaired outcome prediction. Future work incorporating diabetes duration and additional metabolic markers, such as insulin resistance or β-cell function, may yield even more accurate prediction models. In addition, the number of observations available at follow-up decreased over longer postoperative periods of time, which may affect the stability and calibration of predictions at later timepoints and should be acknowledged as a limitation. As an autoregressive approach, the framework may be susceptible to error propagation across successive timepoints. However, stable performance across internal and external validation suggests that accumulated error was limited in practice; formal joint modelling may further address this aspect in future work. The largest proportion of the data comes from prospective databases established in Germany, Switzerland, and Sweden. Thus, the data is primarily for a Western European population, which raises questions about external validity. Further validation in more ethnically diverse and socioeconomically varied populations must confirm the model’s applicability in broader settings. Nevertheless, the model had lower variance in predicted BMI than the model developed as part of the SOPHIA trial^15^, whose strength is undoubtedly its high-quality data set from many international randomized clinical trials and databases. There is no information about possible data overlaps with this trial. In addition, the predictions of the present model have not been directly (head-to-head) compared with those of the SOPHIA model.

One of the strengths of the present model is its clinical applicability. The selected features are readily available in most clinical settings and do not require advanced diagnostic procedures. Other measures, such as diabetes duration, are also known to influence diabetes remission^30^.

Interestingly, model training needed a much smaller number of patients than initially thought to deliver a precise prediction, showing machine learning algorithms are able to make predictions based on data from several hundred patients. Improved predictions require a much larger number of patients, especially for patients with a very high BMI, where the deviation in weight loss was much larger. These findings indicate the need for large data sets, ideally international databases, from which such calculations can be made with more exactness.

Regarding a practical approach for selecting procedures from which to calculate predictions, the focus was on the two most commonly performed worldwide^31^. Therefore, newer procedures such as one anastomosis gastric bypass and endoscopic techniques were not included.

Future work should aim to expand the model to include multi-outcome prediction, allowing for comprehensive metabolic risk stratification. A more complete model should also preferably include patient-reported outcomes to complement weight- and metabolic-related outcomes. This study used interpretable ensemble methods; the integration of attention-based architectures, such as TabTransformer, or other deep learning models could further enhance predictive performance, particularly for long-term risk modelling. TabTransformer captures complex feature interactions through self-attention, enabling improved modelling of long-term, non-linear patterns in patient data. Increasing data processing capacities and the evolution of machine learning algorithms should enable us to develop more complex models in the future.

## Supplementary Material

zrag054_Supplementary_Data

## Data Availability

The data sets used and/or analysed during this study and the code are available from the corresponding authors upon reasonable request.
