# Supplementary material for: Development of a predictive model for postoperative body mass index and diabetes outcomes after metabolic bariatric surgery: retrospective cohort study
Source: BJS Open. 2026 Jul 3;10(4):zrag054. doi: 10.1093/bjsopen/zrag054 (PMC13331347; doi:10.1093/bjsopen/zrag054)
Supplement: zrag054_Supplementary_Data [file zrag054_supplementary_data.docx]

**Supplementary Document:**

**Development of a predictive model for postoperative BMI and diabetes mellitus outcomes after metabolic bariatric surgery: Retrospective Cohort Study**

Vincent Ochs^1*^, Lars Kollmann^2*^, Shantanu Roy^1^, Ilan Rosenblum^3^, Adisa Poljo^4,5^, Andreas Heule^1,6^, Bassey Enodien^7^, Maryna Chumakova-Orin^8^, Eric J. DeMaria^8^, Emanuel Burri^9^, Reinhard Stoll^3,^ Otto Kollmar^3^, Robert Rosenberg^3^, Pascal Probst^10^, Markus K. Muller^10^, Stephanie Taha-Mehlitz^4*^, Beat P. Müller^4^, Daniel M. Frey^11^, Piotr Kalinwoski^12^, Marta Przybysz^12^, Mateusz Bartkowiak^12^, Muhammed Said Dalkilic^13^, Abdullah Sisik^13^, Rodrigo Otavio Carvalho De Oliveria^14^, Fatima Martins^14^, Erik Stenberg^15^, Ellen Andersson^16^, Torsten Olbers^16^, Sven Flemming^2^, Florian Seyfried^2^, Florian Ponholzer^17^, Annemarie Weissenbacher^17^, Dietmar Öfner^17^, Johanna Betzler^18^, Mirko Otto^18^, Ralph Peterli^19^, Philippe C. Cattin^1††^, Anas Taha^1,3,8††*^

Collaborators: Mathias Birrer, Mariana Flaifel, Bara Saad, Jasmin Zeindler, Marionna Cathomas, Joël L. Lavanchy, Christina Bogensperger

1. Department of Biomedical Engineering, Faculty of Medicine, University of Basel, Allschwil, Switzerland

2. Department of General, Visceral, Transplantation, Vascular, and Pediatric Surgery, University Hospital Wuerzburg, Wuerzburg, Germany.

3. Department of Visceral Surgery, Cantonal Hospital Baselland, Liestal, Switzerland

4. Clarunis, Department of Visceral Surgery, University Center for Gastrointestinal and Liver Diseases, St. Clara Hospital and University Hospital, Basel, Switzerland

5. Johannes Kepler University Linz, Medical Faculty, Linz, Austria

6. Institute for Medical Informatics, University of Applied Sciences of Northwestern Switzerland, Muttenz, Switzerland

7. Department of Surgery, Cantonal Hospital Glarus, Glarus, Switzerland.

8. Department of Surgery, East Carolina University, Brody School of Medicine, Greenville, NC, USA.

9. University Institute of Internal Medicine, Cantonal Hospital Baselland, Liestal, Switzerland

10. Department of Surgery, Cantonal Hospital Thurgau, Frauenfeld, Switzerland

11. Department of Surgery, Kantonspital Baden, Baden, Switzerland

12. Department of General, Transplant and Liver Surgery, Medical University of Warsaw, Poland.

13. Department of General Surgery, Marmara University Faculty of Medicine, Istanbul, Turkey.

14. iNOVA4Health, NOVA Medical School, Faculty of Medical Sciences, NOVA University of Lisbon, Lisbon, Portugal.

15. Department of Surgery, Faculty of Medicine and Health, Örebro University, Örebro; Sweden.

16. Department of Surgery and Department of Clinical and Experimental Medicine, Linköping University, Norrköping, Sweden.

17. Department of Visceral, Transplant and Thoracic Surgery, Center of Operative Medicine, Medical University of Innsbruck, Innsbruck, Austria.

18. Department of Surgery, University Medical Center Mannheim, Medical Faculty Mannheim, Heidelberg University, Mannheim, Germany.

19. Department Clinical research, University of Basel, Basel, Switzerland

^*^Vincent Ochs and Lars Kollmann equally contributed and share the first authorship

^††^ Anas Taha and Philippe C. Cattin equally contributed and share the last authorship

Corresponding Author:

*Anas Taha, MD

Department of Visceral Surgery, Cantonal Hospital Baselland.

Department of Biomedical Engineering, Faculty of Medicine; University of Basel, Hegenheimermattweg 167C, 4123 Allschwil, Switzerland,

Anas.taha@unibas.ch

Phone: +41 61 207 5402.

Table of Content:

Supplementary eText 1: Participating centers. 4

Supplementary eText 2: Validation design. 4

Supplementary eText 3: Refinement. 4

Supplementary Fig. S1: Flowchart of included patients from all hospitals 5

Supplementary Fig. S2: Decision Curve Analysis (DCA) for the T2D Remission Prediction Model 6

Supplementary Fig. S3: Comparison of Cox proportional hazards models and machine learning framework for predicting Type 2 Diabetes remission 7

Supplementary Fig. S4: Permutation feature importances for the BMI regression model... 8

Supplementary Fig. S5: Permutation feature importances for the T2D remission classification model. 9

Supplementary Fig. S6: Calibration curve of the classification task. 10

Supplementary Fig. S7: Diabetes Mellitus remission probability - stratified by age. 11

Supplementary Table S1: TRIPOD checklist for model development and validation 12

Supplementary Table S2: Baseline characteristics of the data 13

Supplementary Table S3: Baseline characteristics of the external validation data set 16

Supplementary Table S4: Sensitivity analysis of missingness thresholds and model performance 18

Supplementary Table S5: Model parameter of the final regression and classification model 19

Supplementary Table S6: Classification performance evaluation of best model used (for the mean over 10 folds CV and across all clinics) - F1 20

Supplementary Table S7: Comparison of predicted outcomes by surgical intervention in validation cohorts of the best model - RMSE 21

Supplementary Table S8: Comparison of predicted outcomes by surgical intervention in validation cohorts of the best model - F1 21

**Supplementary Text:**

**eText 1: Participating centers**

Data was collected retrospectively from electronic health records of all patients who underwent bariatric surgery at eleven centers across Europe between January 2012 and December 2023. From Sweden, the data was based on anonymous data from the Scandinavian Obesity Surgery Registry, a national research and quality registry including data from nearly all patients undergoing bariatric surgery nationwide. Data from Germany was prospectively collected from the national quality and research registry (StuDoQ) of the German Society of General and Visceral Surgery (DGAV). From Switzerland, data was prospectively collected in the local anonymized databases of the participating centres. Data from Austria, Poland, Portugal and Turkey was retrospectively collected from the local hospital information systems.

This study was carried out across 11 international centers, providing patient data from diverse regions. This collaboration ensured a broad and representative sample for analyzing outcomes of bariatric surgery. The participating centers included:

- **The Clarunis University Center for Gastrointestinal and Liver Diseases, Basel** (Switzerland)
- **The Cantonal Hospital Thurgau** (Switzerland)
- **The GZO Hospital Wetzikon** (Switzerland)
- **The Cantonal Hospital Liestal** (Switzerland)
- **The University Hospital of Würzburg** (Germany)
- **The University Hospital of Mannheim** (Germany)
- **The University Hospital of Innsbruck** (Austria)
- **The Linköping University Hospital** (Sweden)
- **The NOVA University Hospital Lisbon** (Portugal)
- **The Medical University Hospital Warsaw** (Poland)
- **The Marmara Hospital Istanbul** (Turkey)

Each center participated in retrospective data collection, ensuring consistency by standardizing patient demographics, clinical histories, and surgical details across all sites. The dataset included patients who underwent bariatric procedures between 2012 and 2023, creating a robust multicenter foundation for the development and validation of the predictive model the BMI and T2D.

**eText 2: Validation design**

The validation of the model was performed using both internal and external strategies to assess robustness and generalizability.

Internally, a 10-fold cross-validation was conducted across the full dataset (except the holdout test set). This ensured reliable performance estimation and minimized overfitting. In addition, stratified analyses by clinic were performed to understand center-specific behavior and variation.

A leave-one-clinic-out validation approach was implemented to evaluate the model's external validity. In each iteration, the model was trained on data from all clinics except one, which served as an independent holdout test set. This procedure was repeated for each clinic, allowing us to assess how well the model generalized to unseen clinical environments. The results of this external validation are reported in the manuscript and demonstrate the model's transferability across different centers.

In addition, the data of the clinic of Linköping (Sweden) was not used for the CV but kept as an external holdout test set.

**eText 3: Refinement**

SMOTETomek is being used to address class imbalances and is a hybrid resampling technique that combines the Synthetic Minority Over-sampling Technique (SMOTE) with Tomek link removal. SMOTE generates synthetic samples of the minority class by interpolating between existing instances, while Tomek links identify borderline majority class samples, which are then removed to reduce class overlap and improve decision boundary clarity

**Supplementary Figures:**

**Fig. S1: Flowchart of included patients from all hospitals**

Patients’ flowchart

Patients identified n = 11457

**The Clarunis University Center for Gastrointestinal and Liver Diseases, Basel** (Switzerland) n = 1550

**The Cantonal Hospital Thurgau** (Switzerland) n = 738

**The GZO Hospital Wetzikon** (Switzerland) n = 1049

**The Cantonal Hospital Liestal** (Switzerland) = 358

**The University Hospital of Würzburg** (Germany) n = 1169

**The University Hospital of Mannheim** (Germany) n = 1764

**The University Hospital of Innsbruck** (Austria) n = 950

**The Linköping University Hospital** (Sweden) n = 2356

**The NOVA University Hospital Lisbon** (Portugal) n = 105

**The Medical University Hospital Warsaw** (Poland) n = 150

**The Marmara Hospital Istanbul** (Turkyie) n = 1268

Identification

Records excluded n = 1015

Not enough follow up

Screening

Records excluded n = 227

false values

Records screened n = 10442

Records entered in REDCap database n = 10215

Records excluded n = 563

>20% missing data

Inclusion

Records included in analysis
 n = 9652

Follow-Ups

3 months = 9652 2 years = 6555

6 months = 9652 3 years = 3129

12 months = 9652 4 years = 1915

18 months = 9652 5 years = 971

**Fig. S1****: Patient Flowchart****:** The study screened a total of 11457 patients from 11 international centers. These centers provided retrospective data for adult patients undergoing bariatric surgery between January 1, 2020, and December 31, 2023.

Of the 11457 patients identified, 1015 records were excluded due to missing follow-up data. This resulted in 10442 records of which 227 had to be excluded due to values. This resulted in 10215 entries being entered into the REDCap database for further analysis. Additionally, 563 records were excluded because they contained a high percentage of missing data.

After these exclusions, a total of 9652 patient records were included in the final analysis. The final cohort consisted of patients who met all eligibility criteria and had complete data on the key variables necessary for the study's primary and secondary outcomes.

**Fig. S2: Decision Curve Analysis (DCA) for the T2D Remission Prediction Model**


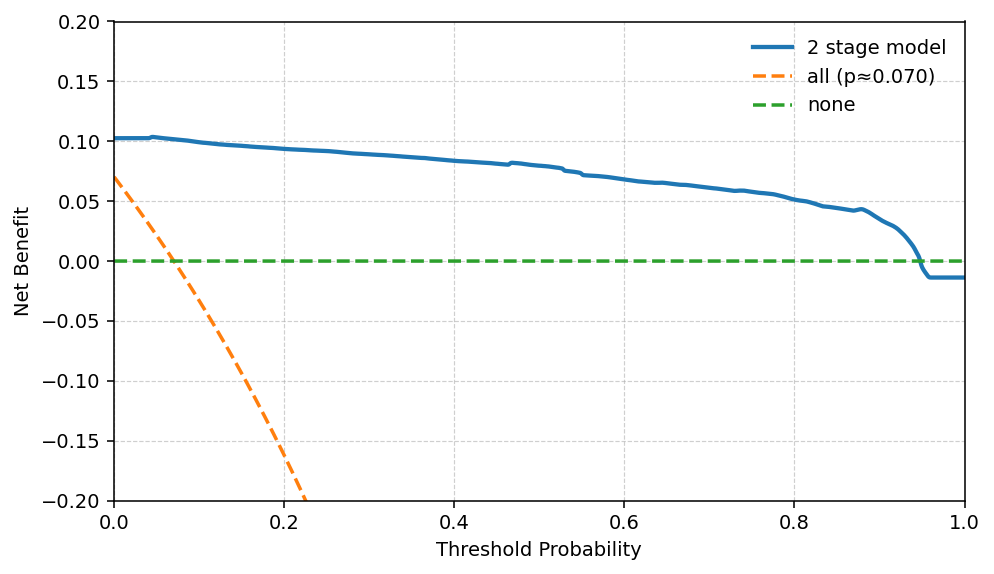


* This figure illustrates the Decision Curve Analysis (DCA) of the two-stage machine learning framework predicting Type 2 Diabetes (T2D) remission after bariatric surgery. The x-axis represents the threshold probability at which a clinician might decide to consider a patient as likely to achieve remission, while the y-axis shows the corresponding net clinical benefit. The blue curve depicts the net benefit of the proposed model, compared to the “treat all” (red dashed) and “treat none” (green dashed) strategies. The model demonstrates a consistently higher net benefit across a broad range of clinically relevant threshold probabilities (approximately 0.1-0.8), indicating that model-guided decision-making yields more true positive identifications of remission without increasing false positives. This suggests meaningful clinical utility of the predictive model in guiding postoperative management decisions.

**Fig. S3: Comparison of Cox proportional hazards models and machine learning framework for predicting Type 2 Diabetes remission**


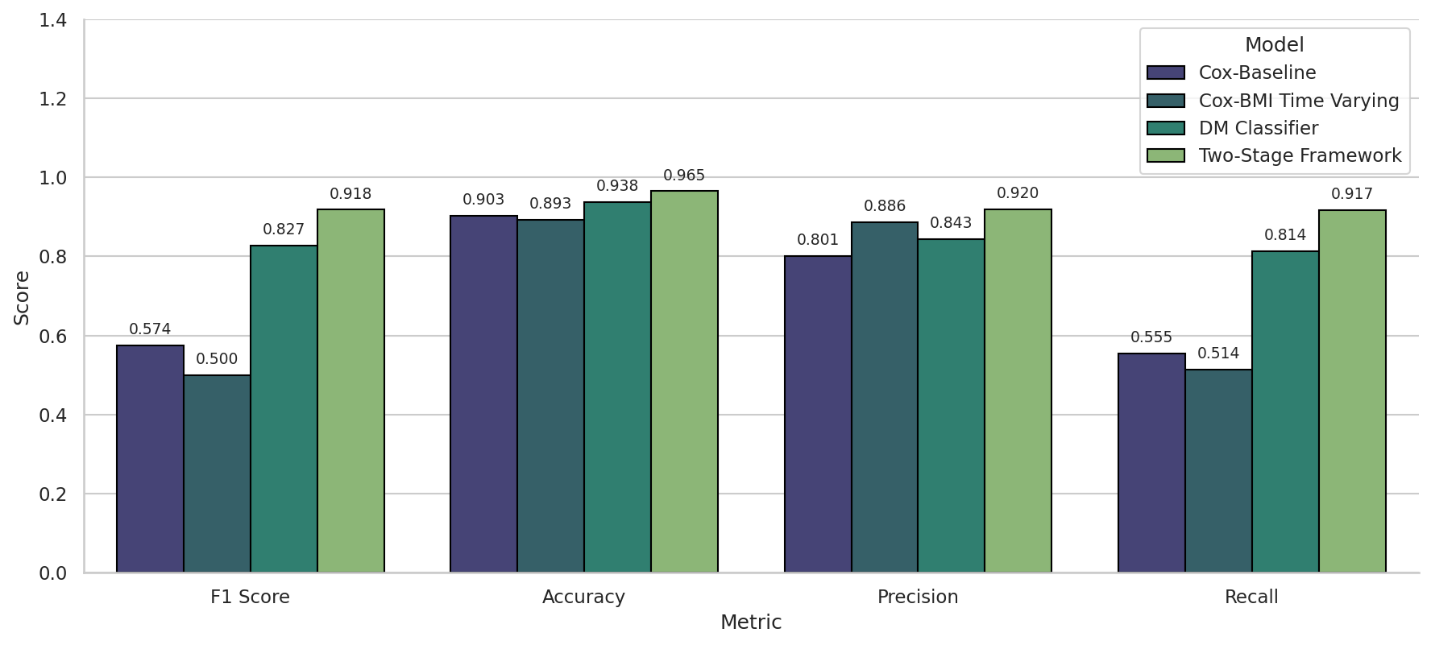


* This figure compares the predictive performance of classical Cox proportional hazards models with the two-stage machine learning (ML) framework for forecasting Type 2 Diabetes (T2D) remission following bariatric surgery. The Cox models include (i) a baseline model using only preoperative clinical variables, and (ii) a model extended with observed postoperative BMI as a time-varying covariate. The ML framework integrates both baseline and dynamically predicted BMI trajectories. The y-axis represents the concordance index (C-index) and area under the ROC curve (AUC) as discrimination metrics across multiple follow-up intervals. The ML approach demonstrates superior discrimination and balanced accuracy compared to Cox models (C-index ≈ 0.93-0.95), confirming improved predictive performance and robustness across time horizons.

**Fig. S4: Permutation feature importances for the BMI regression model.**

**
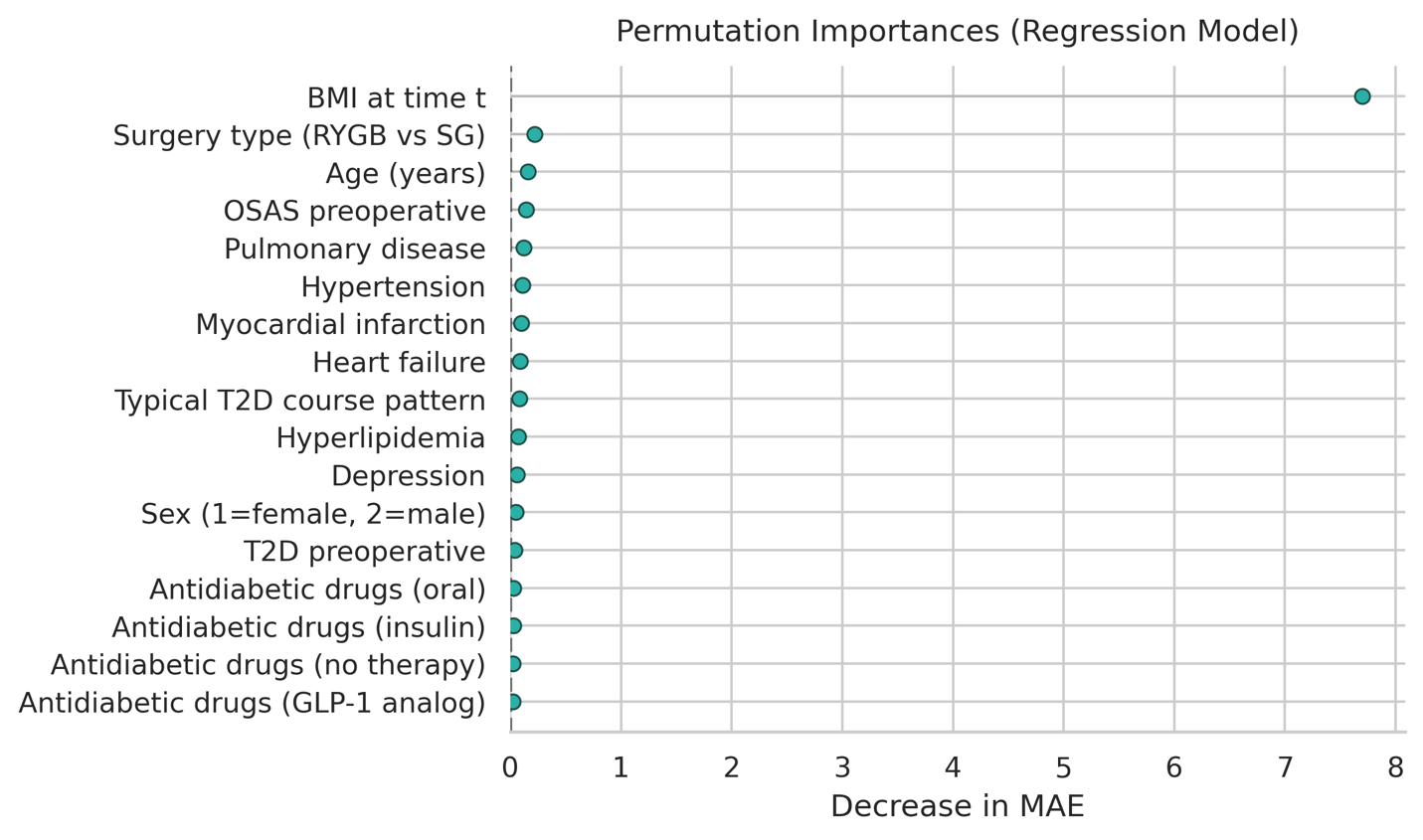
**

* Permutation importance analysis for the postoperative BMI regression model (Hist Gradient Boosting). The x-axis represents the mean decrease in Mean Absolute Error (MAE) after randomly permuting each feature, quantifying its contribution to prediction accuracy. BMI at the current timepoint (*t*) had the highest importance, followed by surgery type (RYGB vs. SG) and age. Other clinical features, including comorbidities and medication use, showed minimal impact on regression performance, confirming that temporal BMI information dominates postoperative BMI prediction.

**Fig. S5: Permutation feature importances for the T2D remission classification model.**

**
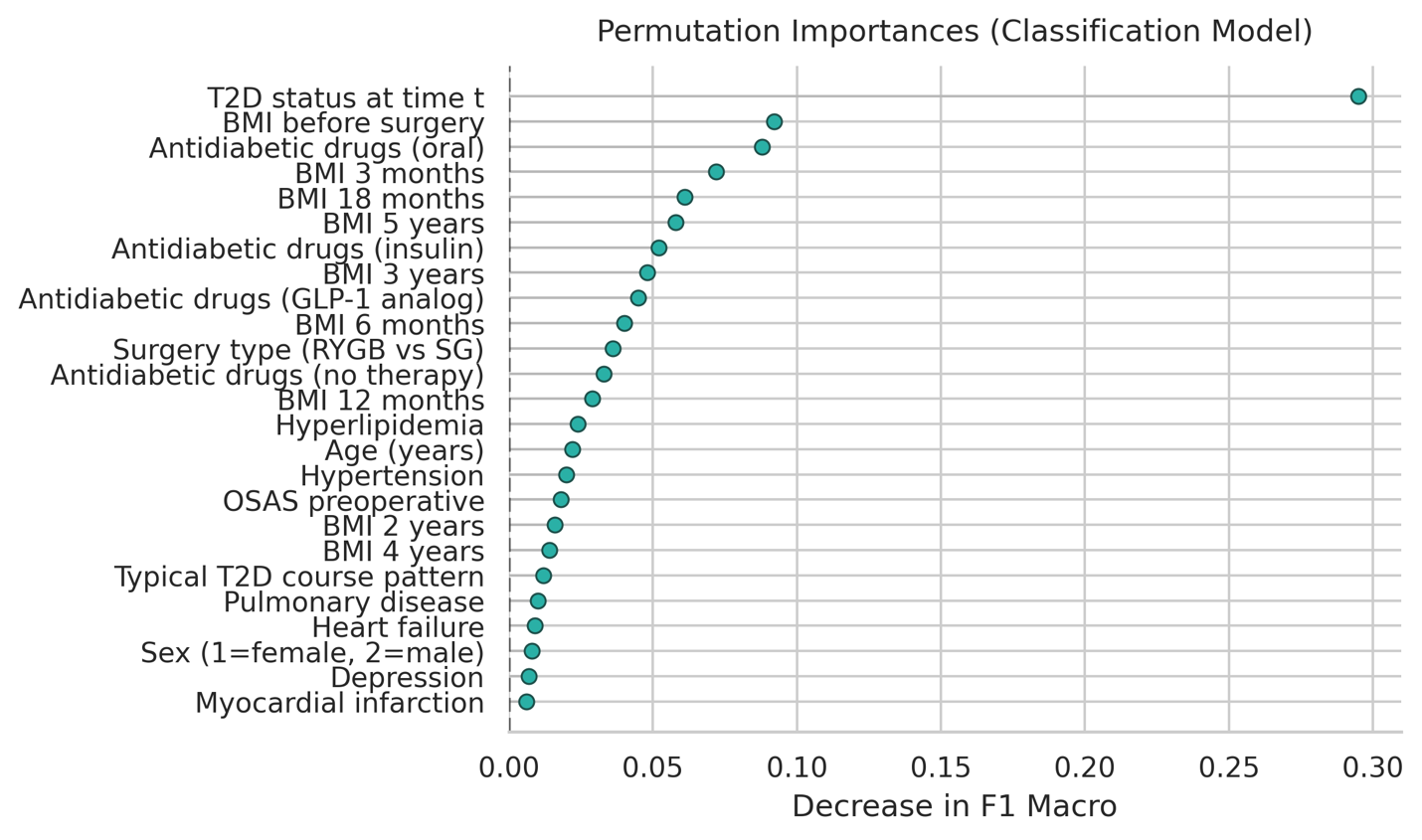
**

* Permutation importance analysis for the T2D remission classification model (XGBoost). The x-axis indicates the mean decrease in macro-averaged F1 score following permutation of each feature. The most influential predictors were current T2D status, preoperative BMI, and antidiabetic medication use (oral and insulin therapy), followed by dynamic BMI estimates at 3-18 months. These findings highlight the clinical relevance of baseline metabolic state and short-term postoperative weight dynamics in driving long-term remission probability.

**Fig. S6: Calibration curve of the classification task.**


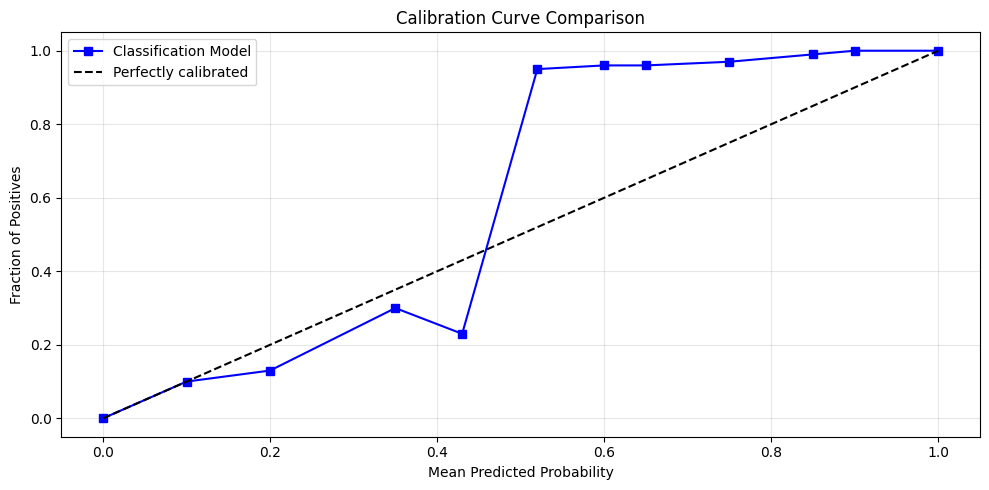


*This figure illustrates the calibration curve of the classification model predicting diabetes remission. The x-axis represents the predicted probability of remission, while the y-axis shows the actual observed proportion of patients achieving remission. Ideally, a perfectly calibrated model would follow the diagonal reference line, indicating that predicted probabilities correspond directly to observed outcomes. In this figure, the blue line represents the model's calibration performance. The curve shows that the model tends to slightly overestimate the probability of remission in the lower prediction range, while predictions in the higher probability range align more closely with actual outcomes. Overall, the model demonstrates reasonable calibration, particularly at higher predicted probabilities, indicating that its probability estimates are generally reliable for clinical interpretation.

**Fig. S7: Diabetes Mellitus remission probability - stratified by age.**


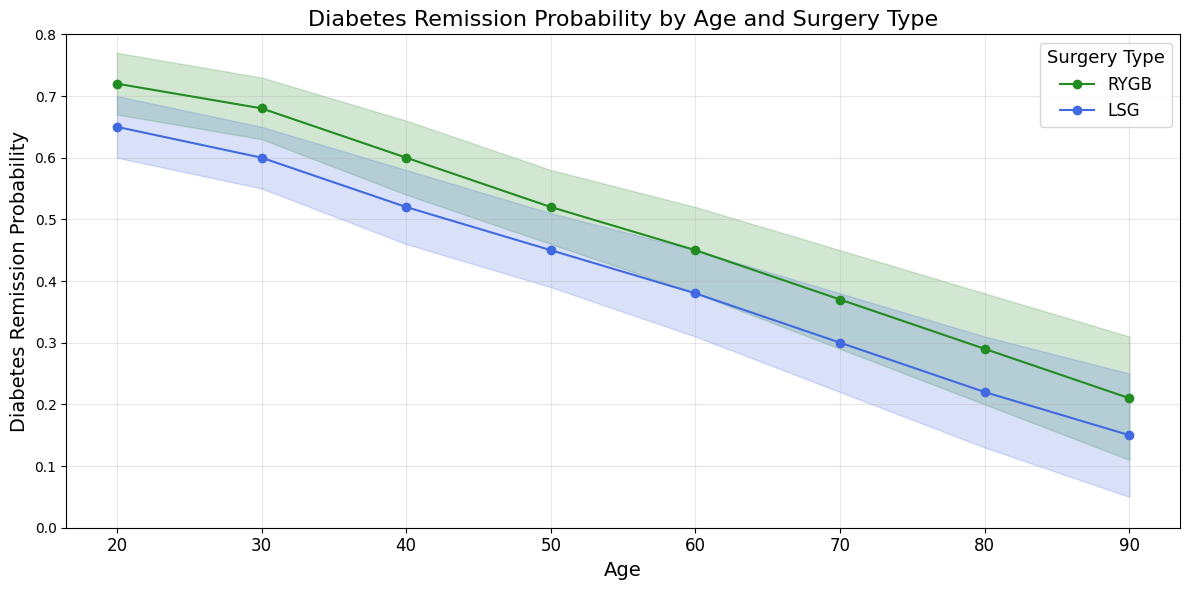


*This figure shows the predicted diabetes remission probability as a function of age for the three bariatric surgery types (RYGB, LSG). Lines with shaded confidence bands represent the predicted remission probability across different age groups, with 95% confidence intervals. Younger patients (aged 20-40 years) show higher predicted remission probabilities, while older patients (above 60-70 years) have markedly lower chances of achieving diabetes remission, independent of surgery type. RYGB demonstrates the highest remission probabilities across all age groups, followed by LSG. This figure illustrates the inverse relationship between age and metabolic improvement, aligning with clinical observations that younger patients benefit more from bariatric surgery in terms of diabetes resolution.

*LSG: Laparoscopic Sleeve Gastrectomy

*RYGB Roux-en-Y Gastric Bypass

**Supplementary Tables:**

**Table S1: TRIPOD checklist for model development and validation**


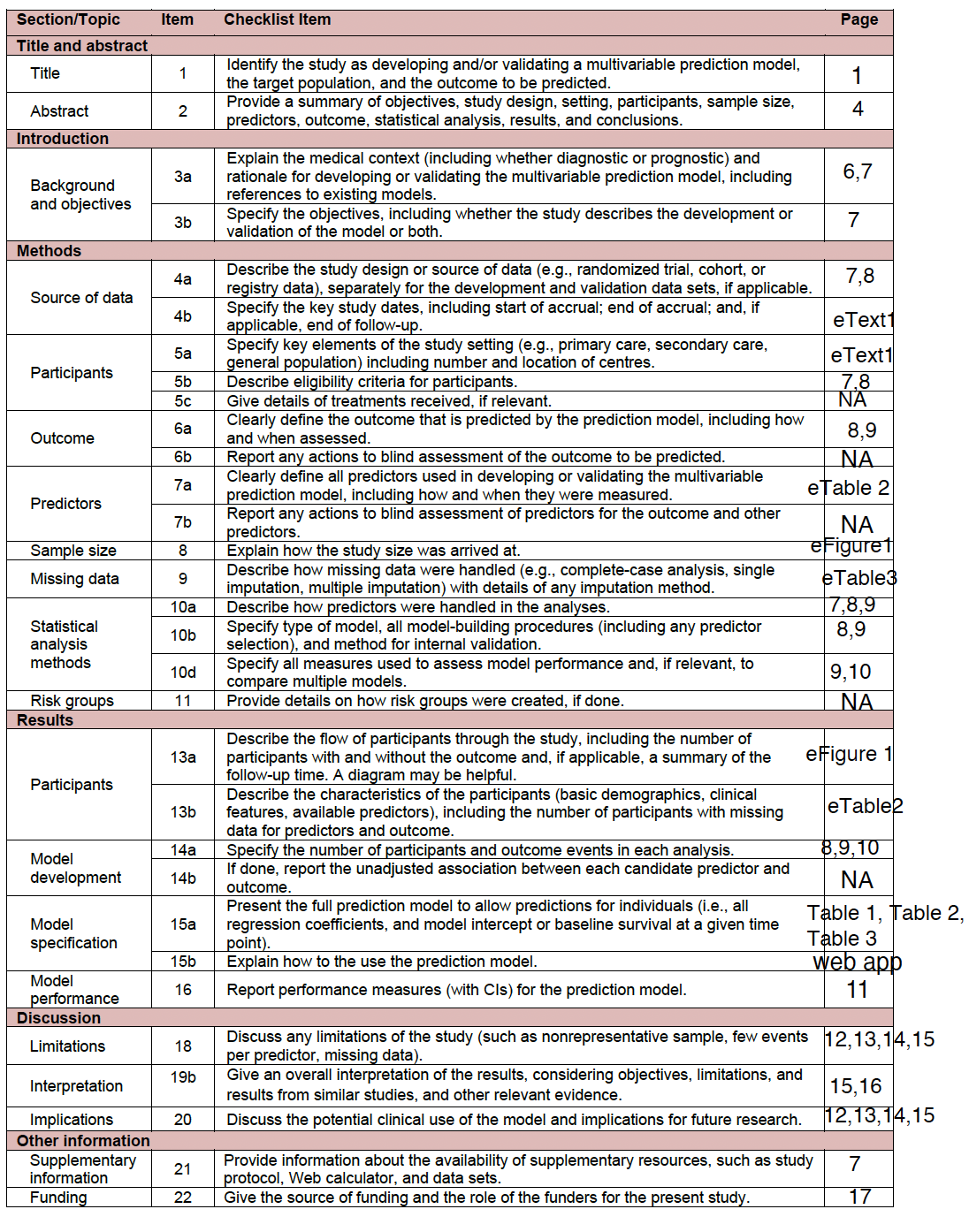


**Table S2: Baseline characteristics of the data**

| **Variable** | **Missing** | **Total**  **n = 9652** | **LSG**  **n = 2852** | **RYGB**  **n = 6800** |
| --- | --- | --- | --- | --- |
| Age | 0.2% | 42.1 ± 11 | 41.7 ± 12 | 42.12 ± 0.4 |
| Sex | 0.1% | NA | NA | NA |
|  | Male | 2423 (25.1%) | 809 (28.3%) | 1614 (23.7%) |
|  | Female | 7219 (74.8%) | 2037 (71.4%) | 5182 (76.2%) |
| ASA | 59% | 1 - 679 (7.0%) | 1 - 387 (13.5%) | 1 - 292 (4.3%) |
|  |  | 2 - 1408 (14.6%) | 2 - 272 (9.5%) | 2 - 1136 (16.7%) |
|  |  | 3 - 1765 (18.2%) | 3 - 637 (22.3%) | 3 - 1128 (16.9%) |
|  |  | 4 - 29 (0.3%) | 4 - 16 (0.5%) | 4 - 13 (0.2%) |
|  |  | 5 - 0 (0.0%) | 5 - 0 (0.0%) | 5 - 0 (0.0%) |
| Charlson Comorbidity Index | 73.8% | 1.0 ± 1.4 | 0.79 ± 1.2 | 1.18 ± 1.4 |
| Hypertension | 29.8% | NA | NA | NA |
|  | No | 3639 (37.7%) | 1199 (42.0%) | 2440 (35.0%) |
|  | Yes | 3133 (32.5%) | 1047 (36.7%) | 2086 (30.7%) |
| Prior abdominal surgery | 28.3% | NA | NA | NA |
|  | No | 2181 (22.6%) | 1044 (36.6%) | 1140 (16.8%) |
|  | Yes | 1817 (18.8%) | 592 (20.7%) | 1225 (18.0%) |
| Hyperlipidemia | 27.3% | NA | NA | NA |
|  | No | 2508 (25.9%) | 1215 (42.6%) | 1293 (19.0%) |
|  | Yes | 1587 (16.4%) | 595 (20.8%) | 992 (14.6%) |
| Depression | 29.5% | NA | NA | NA |
|  | No | 5228 (54.2%) | 1624 (56.9%) | 3604 (50.3%) |
|  | Yes | 1391 (14.4%) | 423 (14.8%) | 968 (14.2%) |
| Anti depression medicine | 96.6% | NA | NA | NA |
|  | No | 191 (1.9%) | 161 (5.6%) | 30 (0.4%) |
|  | Yes | 123 (1.3%) | 38 (1.3%) | 88 (1.3%) |
| Antidiabetic drugs preoperative | 27.1% | NA | NA | NA |
|  | Oral Antidiabetic drugs | 1024 (10.6%) | 309 (10.8%) | 715 (10.5%) |
|  | Insulin | 327 (3.4%) | 100 (3.5%) | 227 (3.3%) |
|  | GLP1-Analogon | 13 (0.1%) | 2 (0.7%) | 11 (0.1%) |
|  | No Therapy | 590 (6.1%) | 238 (8.3%) | 352 (5.2%) |
| OSAS preoperative | 13.1% | NA | NA | NA |
|  | No | 5699 (59.0%) | 1638 (57.4%) | 4061 (59.7%) |
|  | Yes | 2704 (28.0%) | 868 (30.4%) | 1836 (27.0%) |
| GERD preoperative | 96.8% | NA | NA | NA |
|  | No | 160 (1.6%) | 114 (3.9%) | 49 (0.7%) |
|  | Yes | 146 (1.5%) | 117 (4.1%) | 32 (0.5%) |
| Active smoking | 96.8% | NA | NA | NA |
|  | No | 215 (2.2%) | 155 (5.4%) | 60 (0.8%) |
|  | Yes | 93 (0.9%) | 72 (2.5%) | 21 (0.3%) |
| Second operation | 84.6% | NA | NA | NA |
|  | No | 998 (10.3%) | 363 (12.7%) | 635 (9.3%) |
|  | Yes | 493 (5.1%) | 188 (6.6%) | 305 (4.5%) |
| BMI before surgery | 0.0% | 44.24 ± 7.0 | 46.69 ± 8.5 | 43.19 ± 6.0 |
| BMI 3 months | 0.0% | 37.09 ± 6.3 | 39.45 ± 7.7 | 36.30 ± 5.4 |
| BMI 6 months | 0.0% | 33.53 ± 6.1 | 35.75 ± 7.4 | 32.76 ± 5.3 |
| BMI 12 months | 0.0% | 29.93 ± 5.7 | 31.83 ± 6.9 | 29.13 ± 4.9 |
| BMI 18 months | 0.0% | 30.16 ± 6.1 | 33.00 ± 7.2 | 28.87 ± 4.9 |
| BMI 2 years | 0.0% | 29.64 ± 5.7 | 32.39 ± 7.0 | 28.79 ± 4.9 |
| BMI 3 years | 0.0% | 30.76 ± 6.2 | 33.29 ± 7.2 | 29.65 ± 5.3 |
| BMI 4 years | 0.0% | 31.36 ± 6.3 | 33.48 ± 7.4 | 30.39 ± 5.3 |
| BMI 5 years | 0.0% | 31.24 ± 6.0 | 33.29 ± 6.9 | 30.46 ± 5.4 |
| T2D preoperative | 5.2% | NA | NA | NA |
|  | No | 6783 (70.2%) | 1901 (66.6%) | 4882 (71.8%) |
|  | Yes | 2367 (24.5%) | 804 (28.2%) | 1563 (23.0%) |
| T2D 3 months | 43.9% | NA | NA | NA |
|  | No | 971 (10.1%) | 287 (10.1%) | 684 (10.0%) |
|  | Yes | 155 (1.6%) | 54 (1.9%) | 101 (1.5%) |
| T2D 6 months | 43.9% | NA | NA | NA |
|  | No | 1406 (14.5%) | 349 (12.2%) | 1057 (15.5%) |
|  | Yes | 168 (1.7%) | 60 (2.1%) | 108 (1.6%) |
| T2D 12 months | 43.9% | NA | NA | NA |
|  | No | 3896 (40.3%) | 638 (22.4%) | 3258 (47.9%) |
|  | Yes | 435 (4.5%) | 97 (3.4%) | 338 (5.0%) |
| T2D 18 months | 43.9% | NA | NA | NA |
|  | No | 707 (7.3%) | 226 (7.9%) | 418 (6.1%) |
|  | Yes | 108 (1.1%) | 31 (1.1%) | 77 (1.1%) |
| T2D 2 years | 43.9% | NA | NA | NA |
|  | No | 2575 (26.7%) | 488 (17.1%) | 2087 (30.7%) |
|  | Yes | 354 (3.7%) | 76 (2.6%) | 278 (4.1%) |
| T2D 3 years | 43.9% | NA | NA | NA |
|  | No | 873 (9.0%) | 262 (9.2%) | 611 (8.9%) |
|  | Yes | 160 (1.6%) | 51 (1.8%) | 109 (1.6%) |
| T2D 4 years | 43.9% | NA | NA | NA |
|  | No | 751 (7.8%) | 225 (7.9%) | 526 (7.7%) |
|  | Yes | 141 (1.5%) | 43 (1.5%) | 98 (1.4%) |
| T2D 5 years | 43.9% | NA | NA | NA |
|  | No | 1910 (19.8%) | 344 (12.1%) | 1566 (23.0%) |
|  | Yes | 258 (2.7%) | 55 (1.9%) | 203 (3.0%) |

**Table S2: Baseline Characteristics of the Data*

*The table above summarizes the baseline characteristics of patients included in the study, categorized into the different approaches. It includes demographic details, preoperative conditions, surgical parameters, and postoperative outcomes.*

*Additionally, the table reports variables with missing data, their respective percentages, and the total number of patients analyzed for each feature.*

*All features with >30% missings were excluded from the final model (except BMI and T2D).*

**LSG: Laparoscopic Sleeve Gastrectomy*

**RYGB: Roux-en-Y Gastric Bypass*

**ASA: American Society of Anesthesiologists (Physical Status Classification)*

**OSAS: Obstructive Sleep Apnea Syndrome*

**GERD: Gastroesophageal Reflux Disease*

**T2D: Type 2 Diabetes Mellitus*

**Table S3: Baseline characteristics of the external validation data set (Linköping)**

| **Variable** | **Missing** | **Total**  **n = 2356** | **LSG**  **n = 209** | **RYGB**  **n = 2147** |
| --- | --- | --- | --- | --- |
| Age | 0.0% | 45.6 ± 8 | 40.3 ± 2 | 45.6 ± 2 |
| Sex | 0.0% | NA | NA | NA |
|  | Male | 1736 (73.6%) | 146 (69.8%) | 1590 (74.1%) |
|  | Female | 620 (26.3%) | 63 (30.1%) | 557 (25.9%) |
| ASA | 100% | NA | NA | NA |
|  |  | NA | NA | NA |
|  |  | NA | NA | NA |
|  |  | NA | NA | NA |
|  |  | NA | NA | NA |
| Charlson Comorbidity Index | 55.3% | 1.0 ± 2.4 | 1.3 ± 0.3 | 1.41 ± 0.5 |
| Hypertension | 0.0% | NA | NA | NA |
|  | No | 759 (32.2%) | 133 (42.0%) | 1446 (35.0%) |
|  | Yes | 1597 (67.8%) | 58 (36.7%) | 701 (30.7%) |
| Prior abdominal surgery | 0.0% | NA | NA | NA |
|  | No | 2000 (84.9%) | 144 (68.9%) | 1856 (86.4%) |
|  | Yes | 356 (15.1%) | 65 (31.1%) | 291 (13.6%) |
| Hyperlipidemia | 0.0% | NA | NA | NA |
|  | No | 1805 (76.6%) | 176 (84.2%) | 1629 (75.9%) |
|  | Yes | 551 (23.4%) | 33 (15.8%) | 518 (14.1%) |
| Depression | 0.0% | NA | NA | NA |
|  | No | 2046 (86.8%) | 189 (90.4%) | 1857 (86.5%) |
|  | Yes | 310 (13.2%) | 20 (9.6%) | 290 (13.5%) |
| Anti depression medicine | 100% | NA | NA | NA |
|  | No | NA | NA | NA |
|  | Yes | NA | NA | NA |
| Antidiabetic drugs preoperative | 17.5% | NA | NA | NA |
|  | Oral Antidiabetic drugs | 855 (36.3%) | 99 (47.3%) | 756 (35.2%) |
|  | Insulin | 378 (16.0%) | 45 (21.5%) | 333 (15.5%) |
|  | GLP1-Analogon | 10 (0.4%) | 0 (0.0%) | 10 (0.5%) |
|  | No Therapy | 701 (29.8%) | 65 (31.1%) | 349 (16.3%) |
| OSAS preoperative | 0.0% | NA | NA | NA |
|  | No | 1850 (78.6%) | 166 (79.4%) | 1684 (78.4%) |
|  | Yes | 506 (21.4%) | 43 (20.6%) | 463 (21.6%) |
| GERD preoperative | 100% | NA | NA | NA |
|  | No | NA | NA | NA |
|  | Yes | NA | NA | NA |
| Active smoking | 100% | NA | NA | NA |
|  | No | NA | NA | NA |
|  | Yes | NA | NA | NA |
| Second operation | 100% | NA | NA | NA |
|  | No | NA | NA | NA |
|  | Yes | NA | NA | NA |
| BMI before surgery | 0.0% | 42.87 ± 6.0 | 40.45 ± 5.9 | 46.05 ± 0.9 |
| BMI 3 months | 0.0% | 35.54 ± 5.5 | 34.54 ± 5.6 | 39.67 ± 6.0 |
| BMI 6 months | 0.0% | 31.62 ± 5.2 | 31.45 ± 5.1 | 35.63 ± 0.9 |
| BMI 12 months | 0.0% | 29.65 ± 5.3 | 29.98 ± 5.4 | 33.63 ± 0.8 |
| BMI 18 months | 0.0% | 27.69 ± 5.9 | 28.46 ± 5.4 | 31.62 ± 0.9 |
| BMI 2 years | 0.0% | 27.69 ± 5.5 | 28.45 ± 0.9 | 31.63 ± 0.9 |
| BMI 3 years | 0.0% | 28.41 ± 5.6 | 29.43 ± 5.4 | 32.30 ± 1.0 |
| BMI 4 years | 0.0% | 28.63 ± 5.6 | 29.76 ± 5.4 | 32.50 ± 0.9 |
| BMI 5 years | 0.0% | 28.63 ± 5.6 | 29.75 ± 5.4 | 32.52 ± 1.4 |
| T2D preoperative | 0.0% | NA | NA | NA |
|  | No | 1989 (84.4%) | 173 (82.7%) | 2087 (88.6%) |
|  | Yes | 367 (15.6%) | 36 (17.3%) | 60 (2.5%) |
| T2D 3 months | 0.0% | NA | NA | NA |
|  | No | 2151 (91.3%) | 174 (83.2%) | 2090 (97.3%) |
|  | Yes | 205 (8.7%) | 35 (16.8%) | 57 (6.7%) |
| T2D 6 months | 0.0% | NA | NA | NA |
|  | No | 2265 (96.1%) | 182 (87.1%) | 2091 (97.3%) |
|  | Yes | 91 (3.9%) | 27 (12.9%) | 56 (6.7%) |
| T2D 12 months | 0.0% | NA | NA | NA |
|  | No | 2198 (93.3%) | 180 (86.1%) | 2091 (97.3%) |
|  | Yes | 158 (6.7%) | 29 (13.9%) | 56 (6.7%) |
| T2D 18 months | 0.0% | NA | NA | NA |
|  | No | 2173 (92.2%) | 176 (84.2%) | 2090 (97.3%) |
|  | Yes | 183 (7.8%) | 33 (15.8%) | 57 (6.7%) |
| T2D 2 years | 0.0% | NA | NA | NA |
|  | No | 2119 (89.9%) | 175 (83.7%) | 2089 (97.3%) |
|  | Yes | 237 (10.1%) | 34 (16.3%) | 58 (6.7%) |
| T2D 3 years | 0.0% | NA | NA | NA |
|  | No | 2136 (90.6%) | 175 (83.7%) | 2089 (97.3%) |
|  | Yes | 220 (9.4%) | 34 (16.3%) | 58 (6.7%) |
| T2D 4 years | 0.0% | NA | NA | NA |
|  | No | 2184 (92.7%) | 175 (83.7%) | 2091 (97.3%) |
|  | Yes | 172 (7.3%) | 34 (16.3%) | 56 (6.7%) |
| T2D 5 years | 0.0% | NA | NA | NA |
|  | No | 2222 (94.3%) | 177 (84.7%) | 2091 (97.3%) |
|  | Yes | 134 (5.7%) | 32 (16.3%) | 56 (6.7%) |

**Table S3: Baseline Characteristics of the external validation data set.*

*The table above summarizes the baseline characteristics of patients included in the study, categorized into the different approaches. It includes demographic details, preoperative conditions, surgical parameters, and postoperative outcomes.*

*Additionally, the table reports variables with missing data, their respective percentages, and the total number of patients analyzed for each feature.*

*All features with >30% missings were excluded from the final model (except BMI and T2D).*

**LSG: Laparoscopic Sleeve Gastrectomy*

**RYGB: Roux-en-Y Gastric Bypass*

**ASA: American Society of Anesthesiologists (Physical Status Classification)*

**OSAS: Obstructive Sleep Apnea Syndrome*

**GERD: Gastroesophageal Reflux Disease*

**T2D: Type 2 Diabetes Mellitus*

**Table S4: Sensitivity analysis of missingness thresholds and model performance.**

| **Threshold** | **MAE** | **RMSE** | **F1** |
| --- | --- | --- | --- |
| 10% | 0.63 | 1.16 | 0.83 |
| 20% | 0.63 | 1.15 | 0.84 |
| 30% | 0.62 | 1.14 | 0.88 |
| 40% | 0.65 | 1.18 | 0.84 |
| 50% | 0.95 | 1.52 | 0.75 |

*Model performance metrics (MAE, RMSE, and F1 score) are reported for varying exclusion thresholds applied to variables with missing data. Variables exceeding each respective threshold of missingness were excluded before iterative imputation and model training. The 30% threshold yielded the best overall performance, achieving the lowest MAE (0.62) and RMSE (1.14) and the highest F1 score (0.87). Lower thresholds (10-20%) slightly reduced predictive accuracy due to information loss from excessive variable exclusion, while higher thresholds (>40%) introduced greater noise from imputation.

*Variables (predictors) with more than 30% missing values across all patients (e.g., ASA score, Charlson Comorbidity Index, prior abdominal surgery, hyperlipidemia, depression, GERD, smoking, and second operation) were excluded from model training to prevent bias from excessive imputation. A sensitivity analysis was conducted to examine how different variable-exclusion thresholds affected model performance (Table S3). After this variable-level exclusion, patients with more than 20% missing entries across the remaining variables were also excluded (n = 563). Implausible or inconsistent entries (e.g., BMI < 15 kg/m² or > 90 kg/m², duplicate timestamps, negative follow-up intervals) were defined as false values and removed (n = 1242).*

**F1: The F1 Score is the harmonic mean of precision and recall, measuring a model’s accuracy by balancing false positives and false negatives in classification tasks.*

**MAE: Mean absolute error.*

**RMSE: Root mean absolute error.*

**Table S5: Model parameter of the regression and classification model.**

| **Hyperparameter** | **Description** | **Possible Values** | **Value Selected (Regression Model)** | **Value Selected (Classification Model)** |
| --- | --- | --- | --- | --- |
| Learning Rate | The learning rate, also known as shrinkage. This is used as a multiplicative factor for the leaves values. Value of 1 means no shrinkage. | 0.01 / 0.1 / 0.5 / 1.0 | 0.1 | 0.1 |
| Max Depth | The maximum depth of each tree. The depth of a tree is the number of edges to go from the root to the deepest leaf. | 10 / 20 / None | 10 | 10 |
| Min samples leaf | The minimum number of samples per leaf. For small datasets with less than a few hundred samples, it is recommended to lower this value since only very shallow trees would be built. | 1 / 2 / 4 | 2 | 4 |
| L2 regularization | The L2 regularization parameter penalizing leaves with small hessians. Use 0 for no regularization (default). | 0 / 0.5 / 1.0 | 1.0 | 1.0 |

**Table S4: Model parameter of the regression and classification model.*

*This table provides an overview of the hyperparameters used during model optimization for both the regression and classification models in the study. Each hyperparameter controls aspects of how the models learn patterns from data, affecting their complexity, generalization, and performance. Here’s a brief explanation of the listed parameters:*

*Learning Rate:*

*Controls how quickly the model adapts to the problem by scaling leaf node updates. A smaller learning rate (e.g., 0.1) results in slower but more stable learning, reducing the risk of overfitting.*

*Max Depth:*

*Defines how deep the individual decision trees can grow. A limit of 10 prevents overly complex trees, promoting generalizability while capturing meaningful interactions.*

*Min Samples Leaf:*

*Sets the minimum number of samples required at a tree leaf. This acts as a regularization parameter, preventing the model from learning overly specific patterns (overfitting). A smaller value allows for finer splits.*

*L2 Regularization:*

*Penalizes overly large leaf weights, reducing model complexity and enhancing robustness, especially useful for preventing overfitting when small sample sizes exist.*

*The “Value Selected” columns specify which hyperparameter settings were ultimately chosen after hyperparameter tuning for each model type:*

*Regression Model: Tuned for predicting BMI trajectories.*

*Classification Model: Tuned for predicting diabetes remission.*

**Table S6: Classification performance evaluation of best model used (for the mean over 10 folds CV and across all clinics) - F1.**

| **Clinic** | **Month 3** | **Month 6** | **Month 12** | **Month 18** | **Year 2** | **Year 3** | **Year 4** | **Year 5** |
| --- | --- | --- | --- | --- | --- | --- | --- | --- |
| Innsbruck | 0.73 | 0.82 | 0.88 | 0.92 | 0.9 | 0.86 | 0.86 | 0.85 |
| Warsaw | 0.75 | 0.82 | 0.88 | 0.94 | 0.96 | 0.98 | 0.9 | 0.85 |
| Wurzburg | 0.74 | 0.8 | 0.83 | 0.86 | 0.91 | 0.87 | 0.87 | 0.84 |
| NOVA Lisbon | 0.81 | 0.78 | 0.95 | 0.96 | 0.98 | 0.97 | 0.93 | 0.87 |
| Mannheim | 0.82 | 0.76 | 0.77 | 0.83 | 0.89 | 0.86 | 0.78 | 0.81 |
| Clarunis | 0.79 | 0.89 | 0.89 | 0.96 | 0.97 | 0.94 | 0.92 | 0.9 |
| Thurgau | 0.77 | 0.76 | 0.81 | 0.82 | 0.86 | 0.86 | 0.85 | 0.81 |
| GZO Wetzikon | 0.77 | 0.87 | 0.9 | 0.94 | 0.95 | 0.93 | 0.92 | 0.9 |
| KSBL Liestal | 0.91 | 0.96 | 0.92 | 0.95 | 1 | 0.99 | 0.95 | 0.91 |
| Marmara Istanbul | 0.87 | 0.86 | 0.84 | 0.92 | 0.96 | 0.96 | 0.87 | 0.85 |

**Table S5 summarizes the F1 performance of the best classification model (XGBoost), averaged over 10-fold cross-validation for each clinic individually. Classification performance was evaluated at regular follow-up intervals from 3 months to 5 years after surgery. The Mean row reflects the overall average F1 score across all clinical sites, indicating consistently high classification performance throughout the follow-up period, particularly after the first year. Overall, the table illustrates that the model reliably distinguishes between patients with and without diabetes remission across clinics and timepoints.*

**F1: The F1 Score is the harmonic mean of precision and recall, measuring a model’s accuracy by balancing false positives and false negatives in classification tasks.*

**CV: Cross Validation*

**Table S7: Comparison of predicted outcomes by surgical procedure in validation cohorts of the best model - RMSE**

| **Procedure** | **Month 3** | **Month 6** | **Month 12** | **Month 18** | **Year 2** | **Year 3** | **Year 4** | **Year 5** |
| --- | --- | --- | --- | --- | --- | --- | --- | --- |
| LSG | \| 1.35 \| \| --- \| | \| 1.35 \| \| --- \| | \| 1.35 \| \| --- \| | \| 1.35 \| \| --- \| | \| 1.35 \| \| --- \| | \| 1.35 \| \| --- \| | \| 1.35 \| \| --- \| | \| 1.35 \| \| --- \| |
| RYGB | \| 1.28 \| \| --- \| | \| 1.28 \| \| --- \| | \| 1.28 \| \| --- \| | \| 1.28 \| \| --- \| | \| 1.28 \| \| --- \| | \| 1.28 \| \| --- \| | \| 1.28 \| \| --- \| | \| 1.28 \| \| --- \| |

*This table summarizes the Root Mean Squared Error (RMSE) values achieved by the best-performing model for predicting postoperative Body Mass Index (BMI) across different surgical interventions (LSG, RYGB) over the follow-up period. RMSE (Root Mean Squared Error) is a commonly used metric to evaluate regression models. It measures the average magnitude of the prediction errors, with lower values indicating better model accuracy. RMSE is expressed in the same units as the predicted variable—in this case, BMI (kg/m²). The table shows RMSE values stratified by surgery type. This comparison allows assessing how well the model performs across procedures and over time. Generally, lower RMSE values suggest more accurate BMI predictions for a given procedure and follow-up interval.

*LSG: Laparoscopic Sleeve Gastrectomy

*RYGB Roux-en-Y Gastric Bypass

**Table S8: Comparison of predicted outcomes by surgical procedure in validation cohorts of the best model - F1**

| **Procedure** | **Month 3** | **Month 6** | **Month 12** | **Month 18** | **Year 2** | **Year 3** | **Year 4** | **Year 5** |
| --- | --- | --- | --- | --- | --- | --- | --- | --- |
| LSG | 0.92 | 0.92 | 0.96 | 0.96 | 0.88 | 0.94 | 0.93 | 0.95 |
| RYGB | 0.86 | 0.85 | 0.95 | 0.96 | 0.91 | 0.93 | 0.96 | 0.96 |

*This table presents the F1 Score achieved by the classification model for predicting diabetes remission across different surgical interventions (LSG, RYGB) at each postoperative follow-up timepoint. The F1 Score is a metric that balances precision and recall, especially useful for evaluating classification models in imbalanced datasets. A score of 1.0 indicates perfect precision and recall, while 0.0 indicates the worst possible performance. Consistently high F1 scores across all procedures and timepoints reflect the model's robust classification performance in predicting diabetes remission outcomes over time.

*LSG: Laparoscopic Sleeve Gastrectomy

*RYGB Roux-en-Y Gastric Bypass
